# Application of the weighted histogram method for calculating the thermodynamic parameters of the formation of oligodeoxyribonucleotide duplexes

**DOI:** 10.18699/VJGB-23-93

**Published:** 2023-12

**Authors:** I.I. Yushin, V.M. Golyshev, D.V. Pyshnyi, A.A. Lomzov

**Affiliations:** Institute of Chemical Biology and Fundamental Medicine of the Siberian Branch of the Russian Academy of Sciences, Novosibirsk, Russia Novosibirsk State University, Novosibirsk, Russia; Institute of Chemical Biology and Fundamental Medicine of the Siberian Branch of the Russian Academy of Sciences, Novosibirsk, Russia Novosibirsk State University, Novosibirsk, Russia; Institute of Chemical Biology and Fundamental Medicine of the Siberian Branch of the Russian Academy of Sciences, Novosibirsk, Russia; Institute of Chemical Biology and Fundamental Medicine of the Siberian Branch of the Russian Academy of Sciences, Novosibirsk, Russia Novosibirsk State University, Novosibirsk, Russia

**Keywords:** DNA, hybridization, thermodynamic parameters, Gibbs free energy, Weighted Histogram Analysis Method, WHAM, molecular dynamics, ДНК, гибридизация, термодинамические параметры, свободная энергия Гиббса, метод взвешенных гистограмм, WHAM, молекулярная динамика

## Abstract

To date, many derivatives and analogs of nucleic acids (NAs) have been developed. Some of them have found uses in scientific research and biomedical applications. Their effective use is based on the data about their properties. Some of the most important physicochemical properties of oligonucleotides are thermodynamic parameters of the formation of their duplexes with DNA and RNA. These parameters can be calculated only for a few NA derivatives: locked NAs, bridged oligonucleotides, and peptide NAs. Existing predictive approaches are based on an analysis of experimental data and the consequent construction of predictive models. The ongoing pilot studies aimed at devising methods for predicting the properties of NAs by computational modeling techniques are based only on knowledge about the structure of oligonucleotides. In this work, we studied the applicability of the weighted histogram analysis method (WHAM) in combination with umbrella sampling to the calculation of thermodynamic parameters of DNA duplex formation (changes in enthalpy ΔH°, entropy ΔS°, and Gibbs free energy ΔG° 37). A procedure was designed involving WHAM for calculating the hybridization properties of oligodeoxyribonucleotides. Optimal parameters for modeling and calculation of thermodynamic parameters were determined. The feasibility of calculation of ΔH°, ΔS°, and ΔG° 37 was demonstrated using a representative sample of 21 oligonucleotides 4–16 nucleotides long with a GC content of 14–100 %. Error of the calculation of the thermodynamic parameters was 11.4, 12.9, and 11.8 % for ΔH°, ΔS°, and ΔG° 37, respectively, and the melting temperature was predicted with an average error of 5.5 °C. Such high accuracy of computations is comparable with the accuracy of the experimental approach and of other methods for calculating the energy of NA duplex formation. In this paper, the use of WHAM for computation of the energy of DNA duplex formation was systematically investigated for the first time. Our results show that a reliable calculation of the hybridization parameters of new NA derivatives is possible, including derivatives not yet synthesized. This work opens up new horizons for a rational design of constructs based on NAs for solving problems in biomedicine and biotechnology.

## Abstract (ru)

На сегодняшний день разработан широкий спектр производных и аналогов нуклеиновых кислот. Некоторые из них нашли применение при решении научно-исследовательских задач и задач биомедицины. Детальная информация о свойствах таких соединений является основой их эффективного использования. Одну из наиболее значимых физико-химических характеристик олигонуклеотидов – термодинамическую стабильность их дуплексов с ДНК и РНК – можно рассчитывать лишь для некоторых производных нуклеиновых кислот: LNA, мостиковых олигонуклеотидов и PNA. Существующие подходы основаны на анализе экспериментальных данных и построении прогностических моделей. Проводятся пилотные исследования, направленные на разработку методов прогнозирования свойств нуклеиновых кислот с использованием методов компьютерного моделирования, основанные только на знании структуры олигомеров. В данной работе исследована применимость метода взвешенных гистограмм (WHAM) при анализе зонтичной выборки для расчета термодинамических параметров формирования ДНК-дуплексов: изменения энтальпии ΔH°, энтропии ΔS° и свободной энергии Гиббса ΔG° 37. Отработана процедура расчета гибридизационных свойств олигодезоксирибонуклеотидов с использованием метода взвешенных гистограмм. Подобраны оптимальные параметры проведения моделирования и расчета термодинамических параметров. На примере представительной выборки из 21 олигонуклеотида длиной от 4 до 16 нт и долей G/C пар от 14 до 100 % показана возможность расчета ΔH°, ΔS° и ΔG° 37. Ошибки расчета термодинамических параметров составляют 11.4, 12.9 и 11.8 % соответственно, а температура плавления прогнозируется со средней ошибкой 5.5 °С. Такая высокая точность расчетов сопоставима с экспериментальной и с другими прогностическими методами расчета энергии комплексообразования. В настоящей работе впервые систематически исследовано применение метода WHAM для расчета энергии формирования ДНК-дуплексов. Полученные результаты показывают потенциальную возможность достоверного расчета гибридизационных свойств новых, в том числе еще не синтезированных производных нуклеиновых кислот. Это открывает новые горизонты для рационального дизайна конструкций на основе нуклеиновых кислот для решения задач биомедицины и биотехнологии.

## Introduction

To date, a wide range of derivatives and analogs of nucleic acids (NAs) have been developed, many of which have found applications in solving research problems and problems of biomedicine (e. g., (Wang et al., 2022)). Their effective use is possible due to the availability of detailed information about their physicochemical, molecular-biological, and biological properties. This information exists only for a limited number of derivatives of NAs such as locked nucleic acids (LNAs) (McTigue et al., 2004), peptide NAs (Griffin, Smith, 1998), phosphorothioate derivatives (Eckstein, 2014), phosphoramidate morpholino oligomers (Summerton, Weller, 1997), and bridged oligonucleotides (Lomzov et al., 2006). The development of approaches to the prediction of the properties of NAs, their analogs, and derivatives is absolutely necessary for rational design of oligonucleotide constructs in all the abovementioned applications. The availability of such approaches will greatly simplify both scientific research involving such compounds and the creation of commercial products, for example, molecular diagnostic systems or therapeutic NAs

One of the key physicochemical properties of NA derivatives is their ability to form (and the efficiency of formation of) complexes with complementary sequences of DNA and RNA. Models have been devised to predictively calculate thermodynamic characteristics of the formation of duplex DNA structures (SantaLucia, Hicks, 2004), of duplex RNA structures (Xia et al., 1998), of hybrid DNA/RNA duplexes (Sugimoto et al., 1995; Banerjee et al., 2020), and of some NAderivatives: LNAs (McTigue et al., 2004), bridged oligonucleotides (Lomzov et al., 2006), and peptide NAs (Griffin, Smith, 1998). Such studies are based on analysis of experimental data about hybridization properties of these oligomers with consequent construction of predictive analytical models. In addition, pilot studies are being conducted that are aimed at designing techniques for reliable estimation of formation energy of NA complexes by computer modeling methods. The latter are promising from the standpoint of development of approaches to a priori prediction of properties of NA derivatives that have not yet been synthesized. In a recent paper, D. Dowerah and coworkers proposed a series of new analogs of LNAs with different linkers between O2′ and C4′ atoms (Dowerah et al., 2023). This work indicates high potential and demand for methods predicting the properties of modified NAs by means of only their chemical structure.

**Fig. 1.**
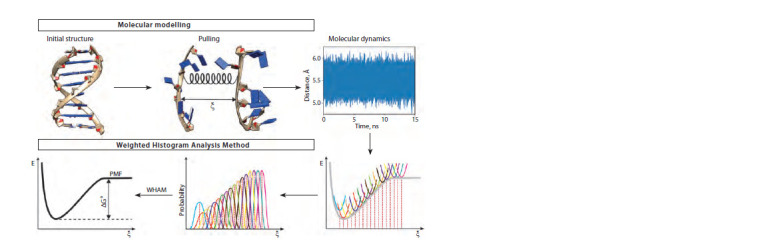
The protocol for calculating the Gibbs free energy of formation of an NA double helix by the WHAM

One well-established approach to the computation of Gibbs free energy is the weighted histogram analysis method (WHAM) combined with an analysis of umbrella sampling (e. g., (Kumar et al., 1992)). The general principle behind this calculation is to carry out molecular modeling by the umbrella sampling procedure and to analyze the resulting trajectories by the WHAM (Fig. 1). In molecular modeling by the umbrella sampling procedure, an additional (usually harmonic) potential is imposed on the system along the reaction coordinate (ξ), and this potential holds the system at position ξi (i = 1 … imax) with a certain force. For each umbrella sampling window (i), a histogram is obtained that represents a probability distribution along the reaction coordinate skewed by the holding potential. One of the most common techniques for calculating the potential of mean force (PMF) from histograms is the WHAM. Within this approach, researchers estimate statistical uncertainty of an unbiased (unshifted) probability distribution taking into account umbrella histograms and then compute the PMF that corresponds to the lowest uncertainty (Kumar et al., 1992). This approach allows to calculate free energy and other observable parameters (Grossfield, 2018).

In this work, we investigated the feasibility of calculating the formation energy of perfect DNA duplexes having various lengths and GC contents by the WHAM coupled with an analysis of an umbrella sample. The computation of the Gibbs free energy of duplex formation at different temperatures should enable us to calculate enthalpy (ΔH°) and entropy (ΔS°) contributions. By means of ΔH° and ΔS° values, it is possible to calculate the most illustrative and widely used characteristic for describing thermal stability of NA complexes: melting temperature (Tm).

##  Methods

The structure of DNA duplexes was created using the NAB program from the AmberTools18 software suite (Case et al., 2018). Starting structures had a B-form of the double helix.

A molecular dynamics (MD) simulation was performed in the AMBER18 software (Case et al., 2018) via parallel com puting on central processing units and graphics accelerators. The ff99bsc0 force field was chosen to model DNA (Pérez et al., 2007). The MD simulation was carried out in an implicit water shell (Tsui, Case, 2000) at a fixed temperature in the range of 273 to 333 K with a step of 10 degrees by means of a Berendsen thermostat with a time constant of 10 ps (Omelyan, Kovalenko, 2013). To enable the step of integration of 2 fs motion equations, we employed the SHAKE algorithm.

The modeling procedure included eight stages

1. Creating the structure of a DNA duplex and saving it in PDB format (with the help of the NAB program from the AmberTools18 software suite). Saving the structure in the amber file format (tleap).

2. Structure minimization for 10,000 steps (pmemd.cuda).

3. Stepwise heating of the system: from 0 to 100 K for 50 ps and from 100 K to a desired temperature (273 to 333 K in steps of 10 K) for 150 ps (pmemd.cuda). An integration time step of 0.5 fs was used

4. Separation of two strands from 0 to 45 Å for 10 ns by applying 10 kcal/mol potential to the distance between the centers of mass of selected atoms of the strands (pmemd.cuda).

5. From the separation trajectory of the two DNA strands, extraction of structures for which the distance between the centers of mass was 0 to 45 (or 60) Å with a step of 0.5 Å (pmemd.cuda).

6. MD simulation of the extracted structures for 15 ns with the imposition of 10 kcal/mol harmonic potential on the distance between the centers of mass of the strands’ selected atoms (pmemd.cuda).

7. Computation of interaction energy of the strands by the WHAM in the WHAM software (Grossfield, 2018). The number of points along the reaction coordinate for sampling of the free-energy profile was set to 150 (see below), and the convergence criterion of the WHAM was 10–6.

8. Calculation of strand interaction energies via componentwise computation of free-energy changes based on MD simulation according to the generalized Born model (molecular mechanics/generalized Born surface area, MMGBSA) was performed using the MMPBSA.py module from the AmberTools18 software suite. Molecular structures were visualized in the UCSF Chimera software (Pettersen et al., 2004).

## Results and discussion

To refine the modeling protocol, a set of DNA oligomers having various lengths (4 to 16 bp) and GC contents (14 to 100 %) was chosen. Nucleotide sequences are given in the Table. The general protocol of the modeling and analysis is presented in Figure 1. We selected an approach where the distance between two DNA strands served as the reaction coordinate. That is, we carried out step-by-step separation of two strands in space and calculated the PMF depending on the distance between them. This approach combined with the WHAM makes it possible to determine the Gibbs energy of interaction between two strands directly in a computational experiment. If such an in silico experiment is conducted at different temperatures, it is possible to calculate a change in the enthalpy and entropy of complexation from a linear dependence of Gibbs free energy on temperature. At the first stage, it is necessary to determine the optimal parameters for performing such calculations.

**Table 1.**
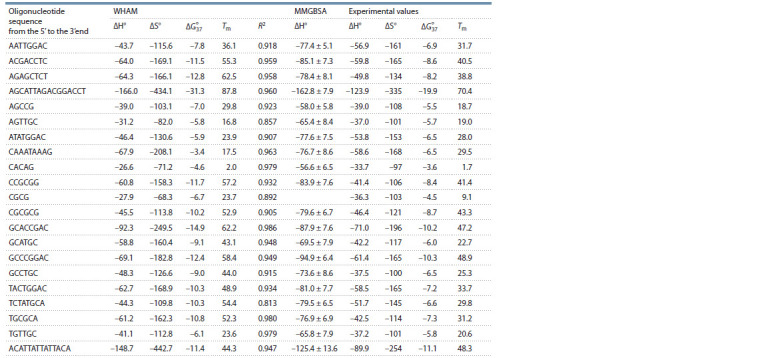
Duplex formation thermodynamic parameters calculated by the WHAM and the MMGBSA method and determined experimentally Note. Sequence only one strand of each duplex is shown. Units of thermodynamic parameters ΔH° and ΔG° 37 : kcal/mol, ΔS°: cal/(mol · K), and Тm: °С. R2: Pearson’s correlation coefficient for linear dependence ΔG°(T). Error of experimental ΔH°, ΔS°, ΔG° 37 and Тm values is 10, 10, 8 %, and 0.5 °С, respectively.

As the reaction coordinate (ξ), we chose the distance (r) between centers of mass of C4′ atoms of all nucleotides from both strands. The initial distance was set to 0 Å in order to (i) examine the possibility of “compression” of the double helix, (ii) determine in the analysis the existence of a minimum of dependence ΔGT° (r), and (iii) compute the energy of complexation as the difference between the minimum and maximum of this dependence (see Fig. 1). Analyzing the separation of the strands’ centers of mass showed that a maximum distance of 45 Å is sufficient for complete dissociation of the duplexes of oligonucleotides with a length of 4 to 9 bp, and for complexes of 14 and 16 bp in size, the distance should be increased to 60 Å. With the reaction coordinate chosen in this manner, the dissociation of the two strands for most oligonucleotide duplexes proceeded in accordance with the unzipping model (Cantor, Schimmel, 1980; Volkov, Solov’yov, 2009), which involves the unwinding of the double helix from one of the ends, or in accordance with a mixed shearing/unzipping mode (Mosayebi et al., 2015; Kurus, Dultsev, 2018). An example of alterations of oligonucleotide conformations along the reaction coordinate is given in Supplementary Material 11. The mechanism of dissociation of duplexes in the current paper is not critically important because only two limiting states are being examined: a relaxed duplex structure and two noninteracting single-stranded oligonucleotides. The match between the helix–coil transition mechanism and the mechanisms observed by experimental methods confirmed the adequacy of the chosen approach for describing the dissociation of a DNA double helix.


Supplementary Materials are available in the online version of the paper: https://vavilov.elpub.ru/jour/manager/files/Suppl_Yushin_Engl_27_7.pdf


According to generally accepted requirements for using the WHAM, it is necessary that the overlap between histograms be at least 20 %. Our analysis indicated that this is 1 Supplementary Materials 1–8 are available at: https://vavilov.elpub.ru/jour/manager/files/Suppl_Yushin_Engl_27_7.pdf achieved at ~0.7 Å between adjacent simulation windows. An example of the dependence of distribution histograms on the distance between strands for duplex 5′-GCACCGAC- 3′/5′-GTCGGTGC-3′ is given in Supplementary Material 2. We chose the reaction coordinate step of 0.5 Å to reliably meet this criterion.

**Fig. 2.**
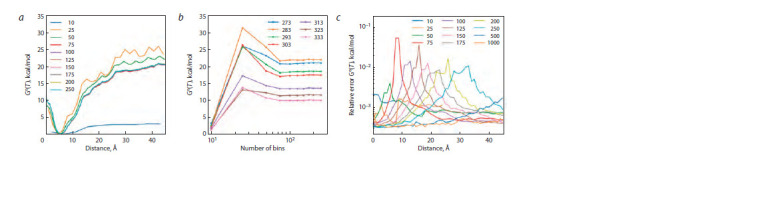
Determination of parameters for the modeling and analysis of MD trajectories. a, The dependence of the Gibbs free energy profile on the distance between the centers of mass of C4’ carbon atoms in two DNA strands for different numbers of points along the reaction coordinate that were chosen to sample the free-energy profile at 273 K. b, The dependence of the Gibbs energy of complexation on the number points along the reaction coordinate that were chosen to sample the free-energy profile. c, The dependence of relative error of Gibbs free energy computation on the distance between the centers of mass of C4’ carbon atoms from the two DNA strands for different numbers of points along the reaction coordinate that were chosen for sampling the free-energy profile.

When energy is calculated by the WHAM, an important parameter is the number of points (“bins”) along the reaction coordinate that are chosen for sampling of the free energy profile. When the number of points was 100 or more (up to 1,000 partitions), a plateau was reached for the shape and position of the Gibbs free energy profiles (Fig. 2, a and Supplementary Material 3) and for the magnitude of the change in Gibbs free energy at different temperatures (see Fig. 2, b). At the same time, relative error values calculated by the bootstrap method (Grossfield, 2018) did not exceed 6 % (see Fig. 2, c).

Gibbs free energy at a certain temperature was calculated as the difference between a minimum and a maximum in the PMF profile: ΔG°(T) = PMFmin – PMFmax. To determine dependence of Gibbs free energy on temperature, the range from 273 to 333 K with a step of 10 K was chosen. The lower value was selected in accordance with the freezing temperature of water and the upper value was chosen to limit the denaturation of an NA duplex in the modeling during the selected time range for short oligonucleotides. This range is wide enough for constructing dependence ΔG°(T) for reliable determination of ΔH° and ΔS° by linear regression analysis via the equation ΔG°(T) = ΔH° – TΔS°.

The trajectory length in the MD simulation at each fixed distance between the selected centers of mass and at a given temperature was set to 15 ns in order to obtain a minimally sufficient trajectory in an implicit water shell for the calculation of thermodynamic parameters (Lomzov et al., 2015). Thus, for each duplex, trajectories 15 ns long were obtained at 90 (or 120) different distances between the centers of mass at seven temperatures. Accordingly, trajectory length for each duplex ranged from 9.45 to 12.6 μs. The total length of trajectories for each complex was more than 200 μs.

**Fig. 3.**
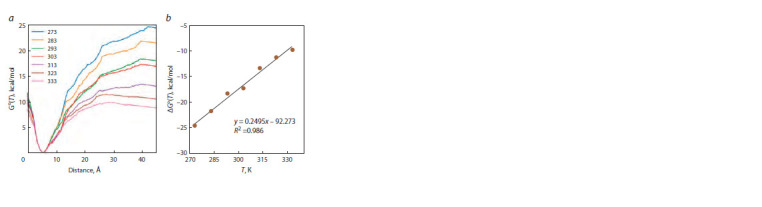
The dependence of Gibbs free energy: a, on the distance between molecules at different temperatures (273, 283, 293, 303, 313, 323, and 333 K); b, on the temperature of model duplex 5’-GCACCGAC-3’/5’-GTCGGTGC-3’.

By the WHAM, the dependence of the Gibbs energy of interaction of two oligonucleotides on the distance (r) between the centers of mass of C4′ atoms of each strand was calculated at seven tested temperatures for 21 studied duplexes (see the Table). A typical dependence of Gibbs energies of complex formation on r at temperatures of 273 to 333 K for the 5′-GCACCGAC-3′/5′-GTCGGTGC-3′ complex is depicted in Figure 3, a. The dependence of Gibbs free energy has a clear-cut minimum near 6 Å and increases with either an approach or dissociation of the double helix strands. During the dissociation, the dependence passes through a maximum and diminishes slightly. The maximum corresponds to the distance at which the interaction between the strands disappears.

To assess the adequacy of the modeling, we compared the geometry of the DNA double helix of the 5′-GCACCGAC- 3′/5′-GTCGGTGC-3′ duplex in a relaxed form with literature data (Supplementary Material 4). All structural parameters are in good agreement with the data on Drew-Dickerson dodecamer (DDD, 5′-CGCGAATTCGCG-3′) structure determined experimentally by NMR spectroscopy (Protein Data Bank [PDB] ID: 1NAJ) and by X-ray crystallography (PDB ID: 1BNA).

Gibbs free energy of complexation was computed at various temperatures. It was established that ΔG°(T) is linear, with a high correlation coefficient R2 of more than 0.83 and an average for all the analyzed complexes of 0.93 (see Fig. 3, b and the Table). Based on the obtained dependences (Supplementary Material 5), changes in the enthalpy and entropy of complexation were calculated next (see the Table). Acomparison of thermodynamic parameters calculated by the WHAM with those determined experimentally (data from (Lomzov et al., 2015)) indicated a linear relation between them with high correlation coefficients R2: 0.87, 0.82, 0.88, and 0.75 for ΔH°, ΔS°, ΔG°37 and Тm, respectively (Supplementary Material 6). As melting temperature, the temperature at which half of oligonucleotides are in a double-stranded state, and the remaining oligonucleotides are in a single-stranded state was chosen. Tm was computed from the thermodynamic parameters (Lomzov, Pyshnyi, 2012) as follows:

**Formula. 1.**
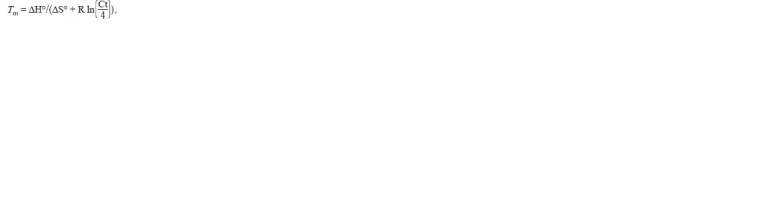
Formula

where R is the universal gas constant, and Ct is the total concentration of oligonucleotides in the system. Ct was set to 10 μM in accordance with typical experimental values.

**Fig. 4.**
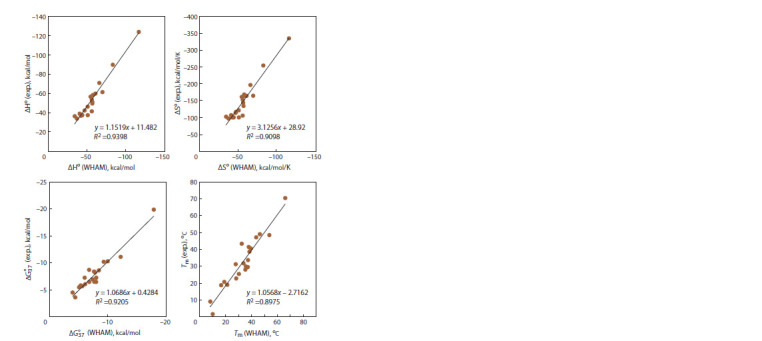
Correlation of thermodynamic parameters ΔH°, ΔS°, ΔG° 37, and melting temperature of complexes – calculated by the WHAM taking into account linear corrections – with experimentally determined parameters (data from (Lomzov et al., 2015)).

The slope of the linear dependence of the thermodynamic parameters was found to be close to 0.5, and values of free terms of the linear dependence are substantial as compared to the analyzed values (see Supplementary Material 6). Therefore, as suggested in our previous papers (Lomzov et al., 2015; Golyshev et al., 2021), it is possible to apply linear corrections to calculated thermodynamic parameters ΔH° and ΔS°. After this correction was applied, correlation coefficients for Gibbs free energy and melting temperatures improved considerably to 0.94 and 0.86, respectively (Fig. 4). In this context, the average absolute error of calculation of thermodynamic parameters became 11.4, 12.9, 11.8 %, and 5.5 °С for ΔH°, ΔS°, ΔG°37 , and Тm, respectively. For our set of oligonucleotides, such error values for thermodynamic characteristics that have been obtained by the MMGBSA method in some studies (Lomzov et al., 2015; Golyshev et al., 2021) taking into account linear corrections are slightly lower: 7.6, 11.4, 10.6 %, and 4.3 °С. The accuracy of the computation of thermodynamic parameters in the present work is comparable to the accuracy of the experimental approach and to that of the most common procedure for calculating the efficiency of oligonucleotides hybridization (the nearest-neighbor method): ~10 % for enthalpy and entropy and approximately 8 % for the Gibbs free energy of complexation (SantaLucia, Hicks, 2004; Lomzov et al., 2006).

To further check the quality of the results, the obtained trajectories were analyzed by the MMGBSA method, and the computed values were compared with the data of the WHAM and with values obtained by the MMGBSA method previously (Lomzov et al., 2015). The typical shape of the dependence of MMGBSA-calculated energy on the distance between the centers of mass of the strands’ C4′ atoms proved to be similar to the dependence of Gibbs free energy on the distance depicted in Figure 3, a (Supplementary Material 7). At distances close to the maximum, the energy of formation of a DNA double helix reaches a plateau of zero, indicating the absence of interaction between the strands when this method of trajectory analysis is employed. The bottom of the potential well was observed in the region of 2–7 Å, which matches the relaxed form of the DNA double helix, and its global minimum near 7 Å is close to the minimum observed in Gibbs energy’s dependence determined by the WHAM (see Fig. 3, a and Supplementary Material 7). There is a weak dependence of the complexation energy calculated by the MMGBSA method on temperature, implying a small change in heat capacity, ΔCp. It seems impossible to reliably determine the change in heat capacity by computational experiments owing to large values of calculation error.

The complexation enthalpy values computed in this work and those determined previously correlate well (R2 = 0.97), with a slope close to unity (0.95) and the free term of the linear dependence close to zero (4 kcal/mol) (Supplementary Material 8, a). Additionally, a similar linear correlation was observed between the complexation enthalpy values calculated by the MMGBSA method and those determined by the WHAM in this work. Thus, the MD trajectories obtained in our paper are realistic.

One of the key aspects of the previously researched energy calculation by the MMGBSA method is the uncertainty associated with the structure of the single-stranded state of oligonucleotides. This state was extracted from the MD trajectory of a double helix. Nevertheless, this approach allowed to calculate the enthalpy of complexation with sufficient accuracy. In this work, during analysis by the WHAM, the single-stranded state of oligonucleotides was fairly well discernible in MD trajectories (as far as this can be done within the framework of the implicit water shell approximation and the force field in question). This approach yielded good results when the energy of double-helix formation was computed. Meanwhile, the main advantage of the WHAM is direct calculation of the change in the Gibbs free energy of DNA double-helix formation. This parameter turned out to be linear in our calculations across a wide range of tested temperatures. This finding suggests that the modeling parameters selected by us and those included in the simulation and model analysis describe the physics of both double- and single-stranded DNA rather well. For the latter, this statement is supported by the conformation of oligonucleotides seen during the modeling of strands with a large distance between their centers of mass (see Supplementary Material 1). Oligonucleotides did not remain linear (in contrast to the duplex), and this observation was utilized in the MMGBSA analysis; they did not become completely disordered strands either but retained several heterocyclic bases in a row in stacking interactions. This finding is consistent with the persistent length of single-stranded regions of oligonucleotides, which is several nucleotides (depending on the GC content and on ionic strength of the solution) (e. g., (Chen et al., 2012)). Furthermore, the linear dependence ΔG°(T) observed in the procedure evaluated in the present paper makes it possible to directly calculate complexation entropy

Nonetheless, the newly developed approach is far from perfect. In particular, for more accurate modeling of the structure and dynamics of DNA, it is necessary to employ the most modern force fields and an explicit water shell model. The analysis of force field parameters for such modeling is a separate, rather complicated task. Besides, the use of an explicit water shell greatly increases the complexity and duration of calculations. For instance, the main computational costs are incurred at the stage of MD calculations. For the 9 bp DNA duplex analyzed in detail in the current work, with the implicit water shell, the calculation speed for a modern video card (NVidia GTX 3080) is ~800 ns/day. Therefore, the computation time for one model duplex is 12 days. With an explicit water shell, the periodic cell being modeled will contain approximately 15–20 thousand molecules owing to the maximum distance between strands of 45 Å. This situation will reduce productivity to ~100 ns/day or approximately 3 months in total. In addition, in an explicit water shell, conformational mobility of DNA will significantly decrease, which will require extending trajectory length for each simulation window, thereby leading to higher computational costs. Nevertheless, such a complication seems necessary to improve the reliability/ accuracy of the calculations

Another promising avenue for the development of the proposed approach is its testing on known modified NAs as examples. This testing should answer the question of the applicability of our approach to the rational design of the chemical structure of new NA derivatives not yet chemically synthesized. The answer can help to solve specific problems of biomedicine and biotechnology. Our present analysis shows high potential and feasibility of the WHAM for calculating the formation energy of duplexes of NAs, their analogs, and derivatives.

## Conclusion

A WHAM procedure for computing hybridization properties of oligodeoxyribonucleotides was refined here. Optimal parameters were selected for modeling and calculating thermodynamic parameters of the formation of DNA duplexes. By means of a representative sample of 21 oligonucleotides 4 to 16 nucleotides long with a GC content of 14 to 100 %, we demonstrated that calculating the enthalpy, entropy, and Gibbs free energy of the formation of oligonucleotide complexes by the WHAM is possible when MD trajectories are analyzed using the following reaction coordinate: the distance between the centers of mass of C4′ carbons of the two strands. Alinear dependence of Gibbs free energy on the temperature at which the simulation is performed was documented. This finding enables researchers to compute the enthalpy and entropy of complexation via an analysis of WHAM results. The calculated thermodynamic parameters linearly correlate with experimentally determined values, with a high correlation coefficient R2 (greater than 0.83). With a linear correction of this dependence, the error of calculation of thermodynamic parameters is comparable with the experimental one and amounts to 11.4, 12.9, and 11.8 % for ΔH°, ΔS°, and ΔG°37, while melting temperature is predicted with an average error of 5.5 °C. Thus, the use of the WHAM for calculating the formation energy of DNA duplexes was systematically investigated for the first time. High accuracy of such calculations was demonstrated, which is comparable with the accuracy of experimental and other techniques for computing the energy of complexation

## Conflict of interest

The authors declare no conflict of interest.
